# 3β-Hydroxy-Δ5-steroidal congeners from a column fraction of *Dendronephthya puetteri* attenuate LPS-induced inflammatory responses in RAW 264.7 macrophages and zebrafish embryo model†

**DOI:** 10.1039/c8ra01967c

**Published:** 2018-05-22

**Authors:** I. P. Shanura Fernando, Won Woo Lee, Thilina U. Jayawardena, Min-Cheol Kang, Yong-Seok Ann, Chang-ik Ko, Young Jin Park, You-Jin Jeon

**Affiliations:** Department of Marine Life Science, Jeju National University Jeju 63243 Republic of Korea youjin2014@gmail.com +82-64-754-3475 +821-045723624; Choung Ryong Fisheries Co. Ltd. 7825, Iljudong-ro, Namwon-eup, Seogwipo-si Jeju-do Korea; Department of Family Medicine, College of Medicine, Dong-A University Busan 602-715 Republic of Korea

## Abstract

Bioactive compounds from marine organisms and their action mechanisms have provided new insights into medicinal and natural product research. Here, we report the identification of 3β-hydroxy-Δ5-steroidal congeners from a purified column fraction (DPCMH24) of the soft coral *Dendronephthya puetteri* harvested from Jeju, South Korea. DPCMH24 exerted strong anti-inflammatory effects through a dose-dependent decrease in the levels of nitric oxide (NO) in LPS-induced RAW 264.7 macrophages (IC_50_ value = 6.54 ± 0.38 μg mL^−1^). Further, DPCMH24 attenuated the levels of PGE_2_ and the pro-inflammatory cytokines, TNF-α, IL-1β, and IL-6. The above effects were mediated *via* the inhibition of nuclear factor κB activation and mitogen-activated protein kinase pathways. *In vivo* evaluation indicated that DPCMH24 reduced NO, iNOS, COX-2, ROS production and cell death in LPS-induced zebrafish embryos, confirming its anti-inflammatory potential. The constituent compounds were identified by GC-MS/MS analysis. These findings suggest that the steroidal congeners from *D. puetteri* may offer ample therapeutic potential against LPS-induced inflammation.

## Abstract 2

Bioactive compounds from marine organisms and their action mechanisms have provided new insights into medicinal and natural product research.

## Graphical abstract

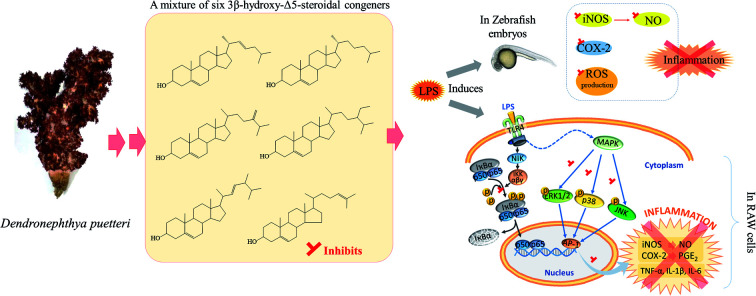

## Introduction

Natural products from marine invertebrates have emerged as a focus for investigation owing to their broad spectrum of bioactive functionalities and unusual structural properties. Soft corals are marine invertebrates thriving in nutrient-rich waters. They are renowned for their constituent terpenoids with notable bioactivity and structural diversity. Many of the steroidal derivatives found in soft corals have received special attention for their antiviral, antimicrobial, cytotoxic, and anti-inflammatory properties. These steroidal derivatives can be found as 1- or 4-en-3-ones, 1,4-dien-3-ones, 9,10- or 9,11-secosteroids, polyhydroxysteroids, steroid peroxides, spiro acetals, hemiacetals, steroidal glycosides, and those with side chains, which often contain the cyclopropane functional group.^1^ Soft corals belonging to the genus *Dendronephthya* are among the most abundant and widespread species. Some of the *Dendronephthya* species are widely exploited for their bioactive natural products. Sarma *et al.* provided a comprehensive review on triterpenoids in soft corals.^2^ Although previous studies have reported the anti-inflammatory potential of terpenoids from several *Dendronephthya* species, the present study is the first to report on the natural products found in *Dendronephthya puetteri*.

Chronic inflammation is a detrimental condition that leads to the pathogenesis of diseases such as cancer, inflammatory arthritis, multiple sclerosis, coronary artery diseases, atherosclerosis, obesity, dermatitis, interstitial cystitis, migraines, insulin resistance, and irritable bowel syndrome.^3–6^ Hence, investigation of the anti-inflammatory properties of natural products has become a cornerstone of modern biomedical research. Steroidal derivatives, especially glucocorticosteroids, are well known for their potent anti-inflammatory properties; they have been found to inhibit a wide range of pro-inflammatory cytokines, including tumor necrosis factor (TNF)-α, interleukins (IL)-1, 3, 4, 5, 6, and 8, and granulocyte macrophage colony-stimulating factor (GM-CSF).^7^ In addition, most of the steroidal derivatives can inhibit inflammatory mediators, such as prostaglandins, leukotrienes, and platelet-activating factor (PAF), which contribute to the observed anti-inflammatory activity.^8^ The present study is a continuation of our previous screening studies in which we analyzed the anti-inflammatory potential of ten different soft coral sample crude ethanolic extracts.^9^ Current disclosure report the identification of natural products found in *D. puetteri* which could contribute for the mediation of the anti-inflammatory effects, through the use of LPS-induced RAW 264.7 murine macrophages and *in vivo* zebrafish embryo model.

## Experimental

RAW 264.7 murine macrophages were obtained from the Korean Cell Line Bank (KCLB, Seoul, South Korea). Adult zebrafish (to obtain the eggs) were purchased from a commercial fish seller South Korea. Dulbecco's Modified Eagle's Medium (DMEM), penicillin/streptomycin mixture and fetal bovine serum (FBS) were purchased from GIBCO INC. (NY, USA). LPS from *Salmonella enterica*, 4-amino-5-methylamino-2′,7′-difluorofluorescein diacetate (DAF-FM DA), 2′,7′-2′7′-dichlorodihydrofluorescein diacetate (DCFH2-DA), 3-(4,5-dimethylthiazol-2-yl)-2,5-diphenyltetrazolium bromide (MTT), and acridine orange were purchased from Sigma, Aldrich, USA. Antibodies for Western blot analysis were purchased from Santa Cruz Biotechnology (USA). ELISA kits (PGE_2_, TNF-α, Mouse IL-1beta, and Mouse IL-6) were purchased from BD Biosciences (USA), eBioscience, Inc. (USA), and R&D Systems, Inc. All the solvents used during the extraction and fractionation were of analytical grade.

### Sample collection and extraction

*D. puetteri* soft coral samples were collected from the Jeju island coast at a depth of 12–15 m during August 2016. The sample was identified by the Jeju Biodiversity Research Institute, and a voucher specimen, JSC16003 was deposited in the laboratory of marine bioresource technology, Jeju National University. The samples were rinsed with tap water to remove attached debris and ground to a powder after lyophilization. 1.2 kg of the powder was extracted three times using a solvent system of 1 : 1 chloroform and methanol. The solvent was evaporated to dryness by a rotary evaporator obtaining the crude extract (DPCM).

### Purification of active fractions by solvent/solvent partition and by open column purification

DPCM was homogenized in water and consecutively fractionated with hexane, chloroform and ethyl acetate obtaining four fractions hexane (DPCMH), chloroform (DPCMC), ethyl acetate (DPCME) and water (DPCMW). The selected DPCMH fraction was further purified using an open silica column with stepwise elution of hexane-ethyl acetate mixture (1 : 0 → 4 : 1 → 3 : 2 → 3 : 7 → 0 : 1). The solvents were removed by vacuum. The selected hexane–ethyl acetate = 4 : 1 eluate was further purified on a second silica open column with stepwise elution of hexane–ethyl acetate mixture (1 : 0 → 19 : 1 → 9 : 1 → 17 : 3 → 3 : 1). Each step of purification was monitored by TLC (TLC Silica gel 60 F_254_, Merck, Burlington, USA) mobile phase hexane : ethyl acetate = 7 : 3 and visualized under UV and by 10% sulfuric acid in ethanol staining → heating method.

### Characterization of compounds by GC-MS/MS analysis

The selected DPCMH24 fraction was analyzed by a Shimadzu GCMS-TQ8040 system (Japan). Capillary column; Rtx-5MS fused-silica, column length 30.0 m, internal diameter 0.25 μm. Sample injection; split mode (ratio 2) injector port temperature 280 °C. GC oven program; hold at 260.0 °C for 3 minutes, increase to 320.0 °C at 6.0 °C min^−1^, increasing to 330.0 at 5.0 °C min^−1^ and hold for 5 minutes. Carrier gas; helium at a constant flow rate (0.73 mL min^−1^).^10^

### Cell-culture

RAW 264.7 mouse macrophages were cultured and maintained in DMEM media supplemented with 10% FBS and 1% penicillin/streptomycin mixture at 37 °C in a humidified atmosphere with 5% CO_2_. The cells were subcultured periodically, and the cells at exponential growth were seeded for the experiments. The samples for the cell culture experiments were prepared by first dissolving the samples in dimethyl sulfoxide (DMSO). Necessary dilutions were carried out using phosphate-buffered saline. The final DMSO concentration in the culture medium after treatment were less than 0.0025%.^11^

### Evaluating the suppressive effects of samples on NO production, and viability of RAW 264.7 macrophages

RAW 264.7 macrophages were seeded (1 × 10^5^ cells per mL concentration) and incubated for 24 h in 24 well plates. Different sample concentrations were treated to the wells and after 1 h, the cells were stimulated by adding LPS (1 μg mL^−1^). Following a 24 h incubation period, a portion of the culture media from each well was withdrawn to a 96 well plate and mixed with a similar volume of Griess regent. NO levels were evaluated by measuring the absorbance at 540 nm. Simultaneously MTT assay was carried out to determine the cell viability.^11^

### Evaluating the levels of PGE_2_ and pro-inflammatory cytokines

Culture media collected from the above-mentioned sample treated, LPS-stimulated wells were used for the determination of PGE_2_ and pro-inflammatory cytokines including TNF-α, IL-1β, and IL-6. The experiments were carried out using ELISA kits following the manufacturer instructions.

### Western blot analysis

Levels of iNOS, COX-2, NFκB p65, NFκB p50, p44/42 MAPK (Erk1/2), Phospho-p44/42 MAPK (Erk1/2), p38 MAPK, and Phospho-p38 MAPK were determined by Western blot analysis. The cells were seeded in 6 well culture plates (2 × 10^5^ cells per mL) and after 24 h, treated with different concentrations of the samples. Following 1 h the cells were stimulated with LPS. The cells were harvested at two different intervals to evaluate the different levels of protein expressions. For the analysis of upstream signaling proteins (related with NFκB and MAPK proteins), the cells were harvested 15 min after the LPS stimulation whereas the levels of iNOS and COX-2 were evaluated 24 h after the LPS-stimulation. Harvested cells were washed with PBS and lysed using a NE-PER® Nuclear and Cytoplasmic extraction kit (Thermo Scientific, Rockford, USA). The protein levels in each cytoplasmic and nuclear extracts were determined using the BCA protein assay kit, and the standardized proteins (50 μg) were loaded on 10% SDS-polyacrylamide gels. The proteins were resolved by gel electrophoresis and transferred to nitrocellulose membranes. Membranes were blocked with 5% nonfat milk for 2 h and incubated with primary (8 h at 4 °C under gentle shaking) and secondary antibodies (2 h at room temperature). After washing away the excess antibodies using TBST, the bands were visualized using enhanced chemiluminescence (ECL) reagents (Amersham, Arlington Heights, IL, USA).^11^

### *In vivo* zebrafish embryo experiments

After acclimating the adult zebrafish to laboratory condition for two weeks, the embryos were obtained by stimulating the fertilization with the onset of light in the morning. Healthy embryos (15 per group) were transferred to separate wells in 12 well culture plates and mounted in 1.0 mL of embryo media. At 8 h post fertilization, different sample concentrations were treated into the wells. Following 1 h, LPS (10 μg mL^−1^) was treated to the wells. The embryonic media was replaced after 24 h of post-fertilization. At the 3rd day of post fertilization (3dpf), the hatched larvae (three from each sample treatment group) were transfered into 24 well plates, stained with specific fluorescence dyes which includes DCFDA, DAF-FM DA, and acridine orange to respectively identify the ROS production, NO production, and cell viability. The fluorescence images of the larvae were obtained using Lionheart™ FX Automated Microscope system BioTek Instruments, Inc. (Winooski, Vermont, USA) with the 469/525 (GFP) filter in place. The intensity of green color was quantified using the ImageJ software and compared with the mean values of respective controls. The levels of iNOS and COX-2 proteins in the embryos were determined by Western blot analysis. The proteins were obtained by homogenizing the embryos under frozen conditions.^11^ The zebrafish experiments were performed in compliance with the guidelines “Ethical principles and guidelines for the care and use of animals” with the approval of Animal Care and Use Committee of Jeju National University of Republic of Korea.

### Statistical analysis

Data values are expressed as the mean ± standard deviation of at least three independent determinations. Statistical analysis for the comparison of results was carryout using one way ANOVA by Duncan's multiple range test using the SPSS statistics V20 software. *P*-values < 0.05 “*” and *P* < 0.001 “**” was considered as significant.

## Results

### Process of bioassay-guided fractionation, purification, and suppressive effect of DPCMH24 on NO production in LPS-induced RAW 264.7 macrophages

**Fig. 1:**
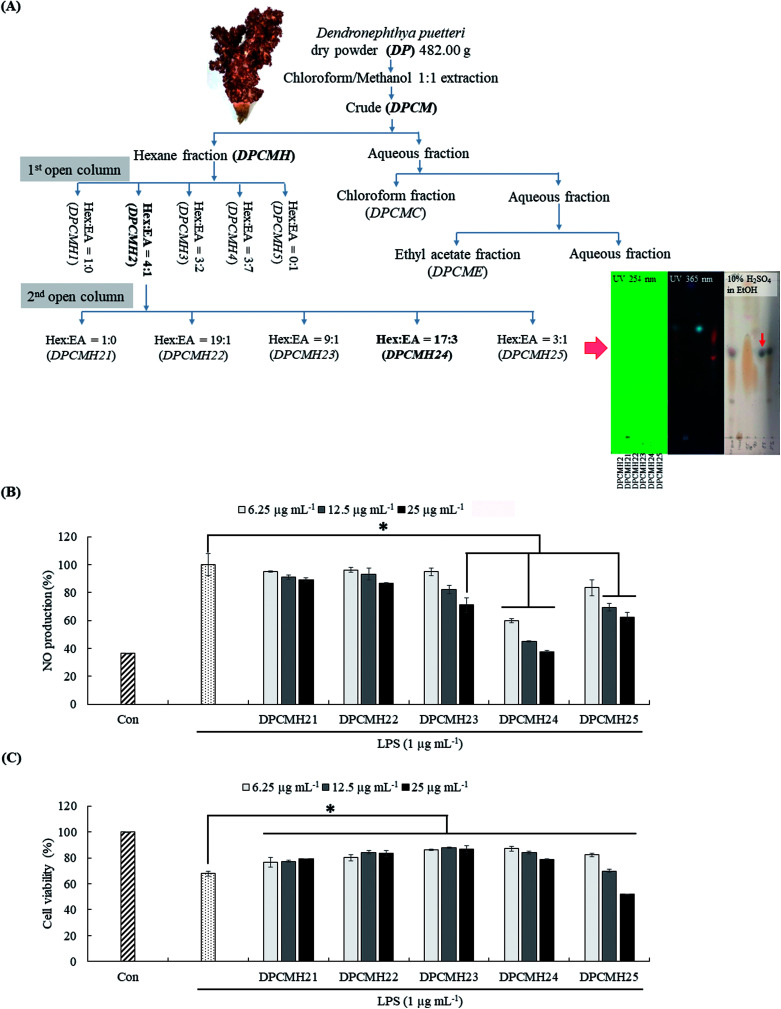
Schematic outline of extraction, bioassay-guided fractionation, and purification process of *D. puetteri* chloroform and methanol 1 : 1 extract, and the effect of DPCMH24 on the reduction in NO production levels in LPS-induced RAW 264.7 macrophages. (A) Purification process, (B) evaluation of NO levels in the cell culture media, and (C) RAW cell viability. The data are expressed as the means ± SE of three replicates in three independent experiments. Significant differences from the positive control (LPS only) group were identified at *P < 0.05 and **P < 0.001.

A graphical summary of the purification process is provided in Fig. 1(A). The chloroform–methanol (1 : 1) solvent system preferably provides a high extraction yield for the terpenoids.^12^ Each step of the purification process was performed after the evaluation of the reduction in NO levels in LPS-stimulated RAW macrophages. Among the solvent/solvent fractionations, the hexane fraction (DPCMH), which was more active, was selected for further purification instead of the chloroform (DPCMC), ethyl acetate (DPCME), and water (DPCMW) fractions. Silica open column purification of DPCMH produced five column fractions, with prominent anti-inflammatory activity and the best cell viability in the second fraction (DPCMH2). DPCMH2 was obtained through elution of the column with a solvent system containing hexane and ethyl acetate, 4 : 1. Further purification of DPCMH2 obtained five column fractions, with prominent anti-inflammatory activity in the fourth column fraction (DPCMH24). Thin-layer chromatography (TLC) analysis revealed that the column fractions were adequately separated. The presence of dark/maroon color spots on TLC, especially in DPCMH24 and DPCMH25 with 10% H_2_SO_4_ in ethanol staining, and their absence under UV light confirmed the presence of sterols. Since reaction with sulfuric acid is highly unspecific, these observations can only suggest presence of sterols. The content of sterols in each of the second column fractions (DPCMH21, DPCMH22, DPCMH23, DPCMH24, and DPCMH25) was 9.60 ± 3.66, 40.79 ± 4.2, 62.07 ± 2.2, 96.49 ± 0.5, and 74.77 ± 1.5% based on the Liebermann–Burchard colorimetric assay, which confirmed the presence of sterols in DPCMH24.

The fractions DPCMH23, DPCMH24, and DPCMH25 reduced NO production in LPS-stimulated RAW macrophages in a dose-dependent manner (Fig. 1(B)). Among the fractions, the most prominent effects were seen in DPCMH24, which had an IC_50_ value of 6.54 ± 0.38 μg mL^−1^. A minor decrease in RAW cell viability was observed with an increase in sample concentrations (Fig. 1(C)). The viability of RAW cells at the IC_50_ value was >85%. As shown in ESI materials (Sheet 2†) the well-known steroidal anti-inflammatory agent dexamethasone indicated an IC_50_ value of 3.39 ± 0.02 μg mL^−1^ for the inhibition of NO production in LPS-stimulated RAW macrophages under similar experimental conditions which was approximately twice stronger than DPCMH24.

### Compounds characterized by DPCMH24 indicated the presence of 3β-hydroxy-Δ5-steroidal congeners

**Fig. 2:**
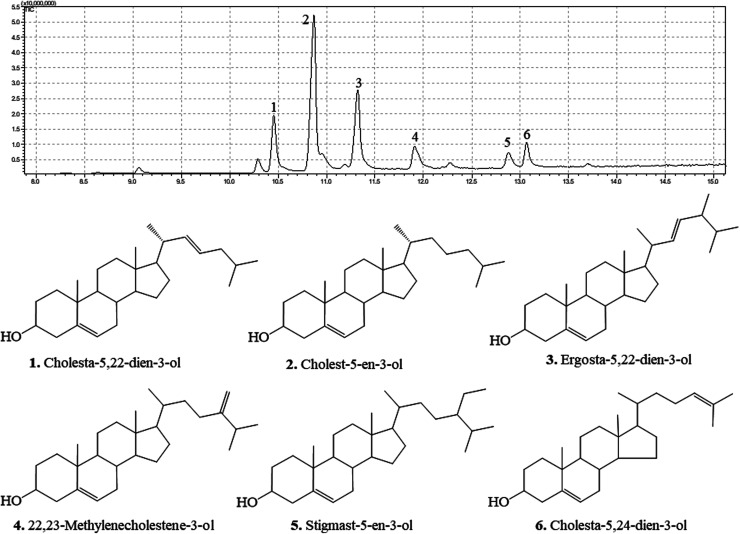
3β-Hydroxy-Δ5-steroidal congeners identified from DPCMH24 by GC-MS/MS. The compounds in DPCMH24 were resolved on a Rtx-5MS fused-silica capillary column and analyzed by using a triple quadrupole mass spectrometer (GCMS-TQ8040). The fragmentation patterns were analyzed by using NIST 17 and Wiley 11 mass spectral libraries.

The GC chromatograms and the identified compounds are presented in Fig. 2. The corresponding mass spectra are provided in the ESI Materials.† DPCMH24 was composed of a mixture of six main 3β-hydroxy-Δ5-steroidal congeners, namely cholesta-5,22-dien-3-ol, cholest-5-en-3-ol, ergosta-5,22-dien-3-ol, 22,23-methylenecholestene-3-ol, stigmast-5-en-3-ol, and cholesta-5,24-dien-3-ol.

### DPCMH24 reduced the production levels of PGE_2_ and pro-inflammatory cytokines in LPS-induced RAW 264.7 macrophages through the inhibition of the NF-κB and MAPK signaling pathways

**Fig. 3:**
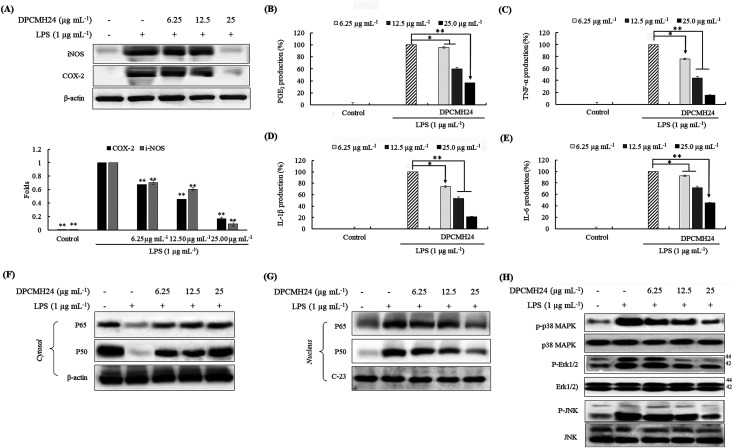
The effects of DPCMH24 on the production of inflammatory mediators, pro-inflammatory cytokines, and NF-κB and MAPK molecular mediators. (A) iNOS and COX-2 levels, (B) PGE_2_ and pro-inflammatory mediators, (C) TNF-α, (D) IL-1β, and (E) IL-6. Analysis of NF-κB and MAPK molecular mediators: (F) cytosolic NF-κB, (G) nuclear NF-κB, and (H) cytosolic MAPK protein levels. Densitometric analysis of the iNOS and COX-2 levels was computed using the ImageJ software. The data are expressed as the means ± SE of three replicates in three independent experiments. *P < 0.05 and **P < 0.001 were significant compared with the positive control (LPS only).

Inflammatory responses are mediated *via* a complex system of signaling pathways. iNOS, COX-2, PGE_2_ are some of the key inflammatory mediators used for the evaluation of anti-inflammatory potential. As shown in Fig. 3(A), treatment with DPCMH24 resulted in a dose-dependent reduction in the levels of iNOS and COX-2 in LPS-induced RAW macrophages. In addition, DPCMH24 dose-dependently reduced the levels of PGE_2_ and the levels of pro-inflammatory cytokines, including TNF-α, IL-1β, and IL-6 (Fig. 3(B)–(E)).

Mitogen-activated protein kinases (MAPKs) and nuclear factor κB (NF-κB) proteins are vital upstream signaling molecules that control the production of inflammatory mediators. Their activation reaches a maximum within 30 min of the LPS-stimulation and decreases thereafter. As shown in Fig. 3(F), the cytosolic levels of NF-κB p50 and p65 in the LPS-treated groups were lower than those in the control group. However, reciprocal events were observed in the nucleus for the levels of NF-κB p50, and p65, where treatment with DPCMH24 dose-dependently reduced their nuclear translocation (Fig. 3(G)). Simultaneous evaluation of IκB levels could have been crucial for justifying/validating the increase in the level of cytoplasmic p50 and p65 with increasing DPCMH24 concentration. The levels of Erk1/2, p38, and JNK were constant in the cytosol of RAW cells, regardless of LPS stimulation or sample treatment (Fig. 3(H)). However, the proportion of phosphorylated Erk1/2, p38, and JNK increased by LPS-stimulation and decreased with the increase in sample concentrations.

### DPCMH24 reduced inflammatory response, in LPS-induced *in vivo* zebrafish embryo model

**Fig. 4:**
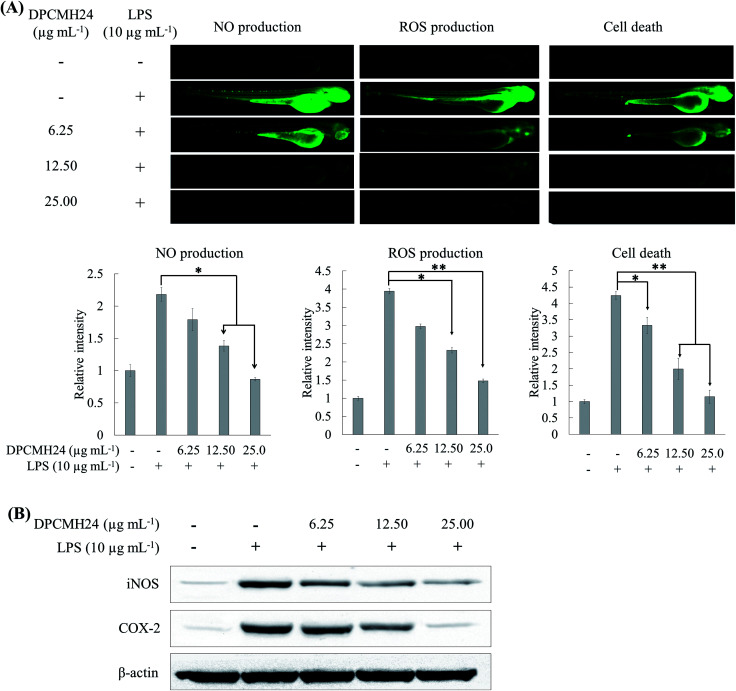
The effect of DPCMH24 on the reduction of inflammatory responses in an LPS-stimulated zebrafish embryo model. Specific fluorescence probe dyes were used to evaluate the reduction in LPS-induced (A) NO, ROS, and cell death. (B) Western blotting of iNOS and COX-2 levels. Quantification of green color fluorescence intensities and the densitometric analysis of Western blotting were computed using ImageJ software. The data are expressed as the means ± SE obtained from three replicates in two independent experiments. *P < 0.05 and **P < 0.001 were considered significant compared to the positive control (LPS only).

Many researchers have focused on the use of zebrafish animal model to mimic a wide variety of human diseases. It is a rapid and sensitive method to screen the anti-inflammatory effects of drug candidates.^13^ In the present study, different fluorescence probe dyes were used to detect NO, ROS, and cell death in the embryos. LPS stimulation caused a marked increase in NO and ROS levels, and cell death in the zebrafish embryos (Fig. 4(A)); however, treatment with DPCMH24 dose-dependently reduced the fluorescence intensity in LPS-induced zebrafish embryos. As shown in Fig. 4(B), density of the Western blotting bands for iNOS and COX-2 increased in LPS-stimulated zebrafish embryos. Treatment with DPCMH24 dose-dependently decreased the iNOS and COX-2 levels in LPS-induced zebrafish embryos. Hence, the observed reduction in NO levels in LPS-induced zebrafish embryos could be attributable to the reduction in iNOS levels.

## Discussion

Inflammation is an essential biological process regulated by complex molecular mechanisms involving inflammatory and anti-inflammatory cytokines, prostaglandins, leukotrienes, growth factors, and nitric oxide (NO). Macrophages are key elements for the regulation of the immune responses triggered by foreign invaders or deregulated biological processes. Activated macrophages cause the production of pro-inflammatory cytokines, such as TNF-α, IL-1β, IL-6, and inflammatory mediators, including prostaglandins (PGE_2_) and NO. Deregulated production of these inflammatory mediators causes pathogenesis of inflammatory diseases such as rheumatoid arthritis, autoimmune diseases, periodontitis, hearing loss, and bacterial sepsis.^14^ Compounds with ability to reduce the levels of these compounds would therefore be promising candidates for the development of anti-inflammatory drugs. As shown in Fig. 1(B), DPCMH24 treatment caused a noticeable reduction in NO production in LPS-stimulated RAW macrophages, with an IC_50_ value of 6.54 ± 0.38 μg mL^−1^. DPCMH24 was found to contain a mixture of six 3β-hydroxy-Δ5-steroidal congeners, namely cholesta-5,22-dien-3-ol, cholest-5-en-3-ol, ergosta-5,22-dien-3-ol, 22,23-methylenecholestene-3-ol, stigmast-5-en-3-ol, and cholesta-5,24-dien-3-ol. In published literature, the reported IC_50_ values of the steroidal and non-steroidal anti-inflammatory drugs dexamethasone and indomethacin are approximately 3.92 μg mL^−1^ and 3.58 μg mL^−1^, respectively.^15^ Hence, the ability of DPCMH24 to reduce NO production in LPS-stimulated RAW macrophages is promising. LPS treatment reduced the viability of RAW cells to approximately 70%, whereas DPCMH24 treatment exerted cytoprotective effects against LPS-induced inflammatory responses and restored cell viability. However, as the concentration of DPCMH24 was increased, minor reductions in cell viability were observed.

An extensive review by Voultsiadou showed that marine invertebrates, including anthozoans, have been used since the ancient Greek and early Byzantium periods for their therapeutic effects. Soft corals are well known for their bioactive sesqui-, tri-, and diterpene metabolites. More than 200 types of steroid metabolites have been identified from soft corals, with a characteristic cholestane structure containing a 3β-hydroxy-Δ5- (or Δ0-) moiety with a side chain of 8–10 carbons.^16^ Carlson *et al.* reported that marine invertebrates contain a wide variety of sterols with unusual structures, some of which are the subject of ongoing investigations.^17^ Byju *et al.* identified six sterols from the soft coral *Subergorgia reticulata* with potential antiproliferative effects, and four compounds (cholesta-5,22-diene-3ol, ergosta-5-22-dien-3ol, cholesterol, and β-sitosterol) were similar to those reported in the present study.^18^ Fernando *et al.* revealed a sterol-rich fraction from *Dendronephthya gigantea* with anti-inflammatory activity (IC_50_ = 4.33 ± 0.50 μg mL^−1^) against NO production in LPS-induced RAW macrophages.^19^ The compounds cholesta-5,22-dien-3-ol, cholest-5-en-3-ol, ergosta-5,22-dien-3-ol, and stigmasta-5,24(28)-dien-3-ol were found, similar to those in the present study. The synergistic effect of these steroidal metabolites might be responsible for the observed anti-inflammatory potential.

Moreover, DPCMH24 dose-dependently inhibited the iNOS, COX-2, and PGE_2_ inflammatory mediators, as well as pro-inflammatory cytokines including TNF-α, IL-1β, and IL-6, which demonstrated a broad range of activity. The observed reduction in NO production could be attributable to the reduction in iNOS levels; moreover, the reduction in COX-2 levels was related to the reduction in PGE_2_. Hence, a detailed investigation was conducted to explore the effect of DPCMH24 on the regulation of upstream signaling molecules that control inflammatory responses.

LPS stimulation causes the activation of the intracellular signaling pathways of MAPKs and NF-κB transcription factors. MAPKs, activated by extracellular stimuli (mitogens), mediate a variety of cellular activities related to cell proliferation, survival, differentiation, and apoptosis; MAPKs include extracellular signal-regulated kinase (Erk), Jun N-terminal kinase (JNK), and p38, and play an essential role in the progression of inflammatory and immune responses. The activation of MAPKs, in conjunction with the activation of NF-κB and interferon regulatory factors, induce the expression of several genes involved in the regulation of the inflammatory responses.^20^ The maximal activation (phosphorylation) of Erk1/2, p38, and JNK was attained 30 min after LPS stimulation and decreased thereafter. However, as a precautionary measure, we harvested the cells 20 min after LPS stimulation. The findings of the present study indicated that LPS treatment increased the phosphorylation of MAPKs, including Erk1/2, p38, and JNK, whereas DPCMH24 treatment suppressed the LPS-induced phosphorylation in a dose-dependent manner. There was no change in the levels of non-phosphorylated MAPKs in RAW cells treated with LPS or LPS and DPCMH24.

The NF-κB family of proteins (p50, p52, p65, c-Rel, and RelB) comprises a set of signaling molecules that are involved in the maintenance of the release of pro-inflammatory cytokines and inflammatory mediators. The phosphorylation of IκB-α proceeds *via* the activation of MAPKs, NF-κB-inducing kinase (NIK), and IκB kinase (IKK).^21^ NF-κB transcription factors, which reside in the cytosol, are composed of dimeric proteins that belong to the Rel homology family (*e.g.*, p50 and p65) and remain bound to the IκB (inhibitor), preventing its nuclear translocation.^22^ Upon activation, phosphorylation of IκB causes the breakdown of the IκB-p50 and p65 complex, which translocates the Rel proteins to the nucleus. Rel proteins bind to κB-binding sites in the target genes to induce the transcription of inflammatory mediators (iNOS, COX-2, and PGE_2_) and pro-inflammatory cytokines (TNF-α, IL-1β, and IL-6).^23^ Many anti-inflammatory drugs can suppress NF-κB activation and consequently inhibit the production of pro-inflammatory cytokines.^24^ In the present study, we observed a decrease in the cytosolic NF-κB p50 and p65 proteins in LPS-treated cells compared to the control. This could be attributed to the phosphorylation of IκB-p50 and p65 complex and translocation into the nucleus. The levels of cytosolic p50 and p65 increased with an increase in the sample concentrations. These observations were in agreement with the results observed for the p50 and p65 levels in the nucleus. The highest levels of p50 and p65 were seen in the LPS-treated group, whereas they were almost absent in the control group. Moreover, reductions in p50 and p65 were observed as the concentrations of DPCMH24 increased. Collectively, these observations suggested that the steroidal congeners in DPCMH24 inhibited the phosphorylation of IκB-p50 and p65 complexes in a concentration-dependent manner.

The LPS-induced *in vivo* zebrafish embryo model is a rapid and sensitive animal model for the investigation of anti-inflammatory properties of drug candidates. The well-developed innate and acquired immune systems in zebrafish, with similarity to mammalian systems, provide an excellent environment to mimic the inflammatory conditions associated with humans. The increased fluorescence intensity of NO and ROS, and cell death in LPS-stimulated zebrafish embryos decreased as the concentrations of DPCMH24 was increased, which suggested that the steroidal congeners in DPCMH24 could act as anti-inflammatory agents. Further, they indicated protective effects against LPS-induced toxicity. Western blotting analysis (Fig. 4(B)) indicated that the iNOS and COX-2 levels were increased in zebrafish embryos after LPS treatment, while DPCMH24 concentrations dose-dependently reduced their levels, supporting its potential anti-inflammatory effects.

In conclusion, the therapeutic potential of the mixture of six 3β-hydroxy-Δ5-steroidal congeners against LPS-induced inflammation offer advantages for their potential applications in the cosmeceutical and pharmaceutical industries. Further studies are required to elucidate the bioactivity of the individual components of DPCMH24 or their synergistic effects.

## Conflicts of interest

There are no conflicts of interest to declare.

## Supplementary Material

RA-008-C8RA01967C-s001
